# Minutes of the Proceedings of the Annual Meeting of the Medical Society of Erie County, N. Y.

**Published:** 1856-02

**Authors:** 


ART. III.—*Minutes of the Proceedings of the Annual Meeting of the Medical Society of Erie County, N. Y.*

*The annual session* of the Medical Society of Erie County, was held in this city, Tuesday, January 8th inst., at the room of the Buffalo Medical Association. There was a full attendance of city members, but a thermometer at zero, a piercing wind, and a driving snow storm, deprived the society of the presence of any of its country members.

The society was called to order at 10½ o'clock, by the *President*, Prof. Jas. P. White, and the proceedings commenced by the reading of the minutes of the semi-annual meeting held in June last, and of two special meetings held in July, all of which were approved by the society.

*Applications* for membership with the society were received from Drs. S. O. Almy, James B. Colgrove, David W. Hershey, Benjamin H. Lemon and William Howell, all of whom after a full examination of their credentials by a committee appointed for that purpose, were admitted to membership, *upon their compliance with the By-laws of the society.*

*The committee* appointed at the last semi-annual meeting upon the application of Dr. D. Devening, for reädmission into the society, made the following report:

The committee appointed at the last regular meeting of this society to investigate the facts relative to Dr. D. Devening, and recommend to this society what disposition should be made of his "application to become a member of this society," would respectfully report:

That we find, upon examination of the records, Dr. Devening was elected a member of the society, after debate, by a majority of 10 votes, in June, 1846.

Soon after being elected to membership, Dr. Devening connected himself in business with his brother-in-law, an irregular practitioner, then, and now, residing in this city. It would seem that Dr. D. was aware that by so doing he violated the rules governing this society, for eighteen months subsequently to his admission, Jan., 1848, he addressed a note to its president asking permission to "withdraw from the society." Whereupon Dr. B. Burwell moved to grant the request, when Dr. Winne moved to lay it upon the table, which carried. In January, 1849, one year subsequently, Dr. Treat moved that

Dr. Devening be expelled, which was adopted, an unsuccessful effort made to amend, and finally referred to a committee of three, consisting of Drs. Treat, Wallace and White. In the following June, more *time* being asked, in compliance with a request of Dr. Devening, in order that he might appear before the committee, it was allowed. In January, 1850, Dr. White, in behalf of the committee, reported, in part, as follows: "That he (Dr. Devening) came before the committee on the 5th instant, that he acknowledges his connection with said Dellenbaugh, is in the same office, and interested with him pecuniarily. That he now expects the connection to cease in the course of a few months, and asks the leniency of the society, but at the same time *denies* the *right* of the society to expel him."

The committee concluded their report by recommending the society "to pursue the course pointed out and prescribed by the laws and regulations of this society," whereupon, on motion of Dr. Josiah Trowbridge, Dr. J. D. Hill acting as teller, the president, after the votes were counted, declared that Dr. Devening was "*unanimously expelled.*" There were present at the meeting on that day, Drs. Wallace, Strong, Newman, King, Lockwood, Barrett, Ring, Williams, Garvin, Pratt, J. S. Trowbridge, Hamilton, Congar, Mixer, Hill, Van Pelt, Lewis, Harvey, Treat, Wyckoff, Hubbard, White, Wilcox, J. Trowbridge, Loomis, Peabody, Cary, Samo, B. Burwell, Pride, G. N. Burwell, Flint, Hauenstein and Barnes; the meeting being, as is perceived, unusually full.

Notwithstanding the protest of Dr. Devening made at that time denying the right of the society to take this action, the committee believe that the society acted in accordance with its powers, and that he was by virtue of that action severed from membership with the society. The same course has been pursued and deemed conclusive in the instances of Stephen Dean, John D. Hill and Noah H. Warner. Your committee, therefore, deem it necessary to elect Dr. Devening to membership provided they desire to renew connection with him.

Your committee would further state that they have ascertained that Dr. Devening has some years since disassociated himself with Dr. Dellenbaugh: that since that time he has deported himself conformably to the rules which govern the profession, and is in good repute, as a practitioner and citizen, with his German brethren and the community. We would add that Dr. D. expresses regret that his course should ever have been such as to meet our disapprobation, and promises conformity to our by-laws and regulations for the future. In view of all the circumstances your committee beg leave to recommend that Dr. Devening be admitted a member of this society upon complying with the by-laws.

James P. White,
H. W. Nelson,
C. C. Wyckoff,
Sanford B. Hunt,
Buffalo, January 8th, 1856. Committee.
To the Erie County Medical Society.

Upon a vote being taken by ballot upon the question of his reädmission, four-fifths of all the members present were found to have voted in favor of the same, so he was again admitted to membership.

*The Dinner Committee* reported that arrangements had been made for the annual dinner at the Clarendon Hotel, at four o'clock in the afternoon.

*The Librarian* made his annual report, and submitted therewith sundry suggestions for the preservation and improvement of his department.

*The Treasurer's Report* gave the resources of the society for the year at $43.74, and the expenditures at $42.99.

On motion of Dr. Hamilton, the following resolution was adopted:

*Resolved,* That the librarian be authorized to procure the volumes of the Transactions of the State Medical Society, and of the American Medical Association necessary to complete the series of each now in the library; and that he continue our subscription for the works of the Sydenham Society; and also that he be authorized to continue the insurance of the library.

Dr. Hutchins delivered the *Oration*: Subject, *Urinary Abscess.*

The thanks of the society were voted him for the same, and a copy requested for preservation.

Dr. G. N. Burwell was appointed *Orator* for the next regular meeting.

Dr. Hunt presented the following *Petition to the Legislature* of this State, with the accompanying *resolution:*

*To the Honorable the Legislature of the State of New York:*

Your Petitioners respectfully represent that the present law for the registration of Births, Marriages and Deaths is inoperative; and that the interests of sanitary reform and medical science are greatly impeded by the want of the statistical information which such a law, if thoroughly carried out, would furnish.

Being convinced that an accurate registration is a matter of great importance in a civilized community, as well with reference to ascertaining the chances, ratio, and average duration of human life, and in furnishing legal proof of heirship, survivorship, and legitimacy of birth, as in assisting the progress of medical science, we do respectfully urge upon your honorable body some such amendment of the present law as shall make it operative.

They further suggest that in confiding its execution to Town and School District Clerks, placing it in the hands of men who have — no penalty being attached for non-compliance — little interest in its fulfillment, and who, moreover, having no similar duties to perform, and little intercourse (officially) with each other, or with the county clerk to whom they report, the present law is wanting in the essentials of success, inasmuch as its execution is placed in improper hands.

Your petitioners, therefore, humbly pray that the execution of the law be placed under the care of the Department of Public Instruction, that the Trustees of all School Districts be obliged to report annually to the Town Superintendent of Common Schools, as a part of their usual annual report, all Births, Marriages and Deaths occurring in their district during the preceding year, under penalty of the forfeiture of their library money for that year; that each Town Superintendent shall be obliged to embody an abstract of the several district reports in his annual report to the county clerk, under penalty of the forfeiture of the library money for his town; and that the county clerk of each county shall embody an abstract of the reports of the towns in his usual annual report to the State Superintendent of Common Schools.

Believing that such a modification of the present law would result in its successful operation, and in great good to the community at large, your petitioners will ever pray.

*Resolved*, That the President and Secretary of this society be directed to sign the said petition in the name and with the seal of the society, and that they forward a copy of it to the members of the Legislature from Erie County, and that our delegates to the State Medical Society be requested to present another copy of the same to the Medical Society of the State of New York, with a respectful request that it should join us in this petition.

*The Petition and Resolution* were adopted by the society, and the charge of the same was conferred to the care of a committee, consisting of Drs. Hunt, Hawley, G. N. Burwell, Lay and Underhill.

Sundry orders were drawn upon the treasurer to cover expenditures and appropriations, and five dollars were voted to the prize fund of the state society.

On motion of Dr. Strong, it was

*Resolved*, That our delegates to the state society be especially charged with the duty of securing for this society a sufficient number of the future volumes of the Transactions of the state society to supply our members.

Dr. Lockwood offered the following resolution, to lie over until the next regular meeting:

*Resolved*, That the fee bill relating to public appointments, adopted at the semi-annual meeting of this society in June, 1854, be and the same is hereby repealed.

The annual election of officers was then held, and choice made of the following gentlemen:

Dr. William Van Pelt, (Williamsville,) *President*,
" Frank H. Hamilton, - - *Vice-President*,
" James M. Newman, - - *Secretary*,
" Charles H. Wilcox, - - *Treasurer*,
" James B. Samo, - - *Librarian*.

Drs. S. Eastman, C. H. Baker and J. S. Hawley, *Primary Board*.

*Censors*.—Dr. P. H. Strong, *Examiner in Anatom., Physiol. and Surgery*,
" C. H. Baker, " " *Practic. Med. and Obstetrics*,
" R. W. Nelson, " " *Chemistry and Pharmacy*,
" C. C. Wyckoff, " " *Mat. Medica and Botany*.
" C. B. Hutchins, " " *Med. Jurisprudence and Path.*

The President then delivered his *Valedictory Address*.

(Dr. White's address is inserted elsewhere in this number.)

At the conclusion of the address, on motion of Dr. Lay, the thanks of the society were presented to the President for his address, and for the faithful courteous and impartial discharge of his duties as its presiding officer during the past year.

On motion of Dr. Newman it was

*Resolved*, That the President's valedictory, or so much of it as relates to the formation of a society for the relief of distressed members of the profession, their widows and children, be referred to a committee to report upon the feasibility of the same, together with any and all such information relative thereto as they may be able to gather, at the next regular meeting.

Said committee was appointed, consisting of Drs. Newman, Harvey and Mixer, to which, on motion of Dr. Mixer, Dr. White's was also added.

Dr. Nelson called up the following resolution prepared by him, June 12th, 1855:

*Resolved*, That in future no applications for membership be received without a certificate from the county clerk accompanying the credentials;

And moved that it be referred to a committee. The motion was adopted, and a committee appointed consisting of Drs. Nelson, Hamilton and Gould.

On motion of Dr. Newman, it was

*Resolved*, That the treasurer be directed to collect all outstanding dues from members,—*peaceably if he can, forcibly if he must.*

The society having transacted all the business before it here, adjourned; but the members reässembled at the Clarendon Hotel, and with a few invited medical guests, at half past four o'clock, sat down to a well filled table. The howling storm without was soon forgotten amid the good cheer and conviviality of the occasion. The only regrets suffered to alloy the festivities, were those felt for absent ones, whose health and prosperity were not forgotten amid the profusion of toasts and sentiments, the spontaneous effusion of the hour. The occasion was one of unalloyed enjoyment, and the members separated resolving to perpetuate the annual dinner.

